# Medical News and Miscellany

**Published:** 1889-03

**Authors:** 


### Medical News and Miscellany.

We welcome the London Lancet to our exchanges.

We Call on our Subscribers to pay up arrears. Some are shamefully behind; start afresh.

Dr. J. J. Mulherron has retired from the the Age, and is succeeded by Prof. B. W. Palmer.

Dr. T. J. Wiggins, of Menardville, was married on the 27th day of February ult. to Miss Dona Redman, of Mason.

The Louisiana State Medical Association wlll meet in the lecture room of Tulane Hall, in New Orleans, April 9 to 11 prox.

Sulfonal seems to be "getting there" as a hypnotic. Prof. Rogers reports favorable results; as also a number of other physicians.

Dr. R. H. Harrison, Jr., of Columbus, Texas, has just recovered from a four weeks spell of sickness. Congratulations of the Journal.

At Paradise, Texas, on the 18th January (last), Dr. J. A. Brown was arrested for forgery. He gave bond of $500, and was released; further particulars not learned.

The Kentucky School of Medicine, at Louisville, Dr. W H. Wathen, Dean, opened the spring session with nearly three hundred students, many Texans amongst them.

Squibb to the Front.—The Kings County Medical Society of New York have elected Dr. E. R. Squibb president, him of "Ephemeris" (al) fame. We presume there was some "squibbling" or "squabbling" before the election.

A Good Appointment.—The New Orleans Polyclinic is to be congratulated upon the acquisition of Dr. R. H. Day to its faculty. Dr. Day has accepted the chair of Diseases of Children, a position he is, by his learning and vast experience, eminently qualified to fill.

THE LATE DR. ROSS, OF BRENHAM.—In our April edition we will publish a biography and portrait of this eminent and good physician whose loss the profession has recently sustained.

DR. J. DUFF BROWN, of Llano, was stricken with paralysis, at his home, on the 18th of January (last), just as he was about to mount his horse to visit a patient. He called in a lawyer and made his will, but at last accounts was still living, the paralysis affecting the lower limbs only.

DR. W. T. RICHMOND'S short but pointed paper on the "management of the cord" which appeared in our January number, has been extensively copied by the medical press. Texas doctors should be encouraged by this fact to contribute their experiences to their home JOURNAL—it does good.

DO NOT, whatever else you do, forget the big meeting to be held at San Antonio, 23d to 26th, next month. Come one, come all, and let's give the grand old State Association a new impetus and renew her lease of life. Five hundred delegates are expected, and a good time generally. The city of the Alamo will have on her Easter dress, all bedecked with flowers.

THE ALABAMA MEDICAL AND SURGICAL AGE, is the name of a neat new medical journal, issued at Anniston, Alabama, by Dr. Jno. C. Legrand. We are pleased to shake hands with Brother Legrand on his accession to the tripod. By the bye, the "Age" claims to be the only medical journal in Alabama. What has become of Brother Davis' journal, at Birmingham?

HIGH OR LOW.—"Is this the right kind of lobelia?" said a lady who had just purchased a vial of that drug. "There is but one kind," said the clerk. "Oh, yes there is; in my garden I have a white and a blue lobelia," said she. "Ah, I see," said the clerk; "the *white* is the *low* belia, and the *blu*e is the *high*

belia.'' (Lady went away pondering on the wisdom of drug clerks in general.)

---

Another Insane Asylum.—The Texas Legislature has appropriated $150,000 to build another insane asylum, to be located '' west of the Colorado.'' As the population of the State has nearly doubled in the last decade, the number of unfortunates has increased, and more room is needed for their accommodation. It is thought it will be located at San Antonio.

---

A Matter for the New Administration to Look to.—Fond mother—''Doctor, what is the matter with my little son?''

Doctor—''Well, Madam, I should say it is some foreign body in his stomach.''

Mother—''Oh dear! those horrid Irish potatoes! I will tell my grocery man that he positively must furnish us some American potatoes!''

---

No Free Medicine in Texas.—The Journal has on several occasions pointed out that under the laws of Texas the State University is required to give medical and law education free, and has insisted that it is an evil which should be corrected. The Twenty-first Legislature has just passed a bill repealing the objectionable clause, and authorizing a charge of $80 per session for instruction in law, and $150 for medicine. Good.

---

Bumpology.—We were commissioned while in New Orleans to buy some books for a friend, and entering a book store, we were confronted by a dude with a 1x3 forehead, and a toothpick in his mouth. We enquired for ''Bump on Bankruptcy.'' Assuming a perplexed but wise look, the dude scanned the shelves a while, then walking slowly toward the rear, he hailed a fellow clerk with, ''I say, Ellis, have we got 'Banks on Bumprumpcy'?''

---

The Governor of Florida called the Legislature in special session to create a State Board of Health. They did so, instanter. The board consists of three members. They will meet, and after selecting a president from among themselves, will select a secretary and an executive, or health officer from the State at large—the latter to be a physician. The board is given full power to protect the State by quarantine or otherwise, as it sees fit.

---

Dr. F. T. Paine says: "That all inflammation is in a state of electro anæsthesia to electricity, and that, therefore, the proper application of electricity to inflamed tissues is entirely painless.

"That electricity so applied to inflamed tissues becomes in turn an anæsthetic, removing all pain, heat, redness, swelling and tension, the essentials of inflammation.

"And thus we hope to have laid the foundation stone of a more correct pathology of inflammation."

---

Appreciative.—We were looking over some old engravings at the old book store, and amongst them was one of Goethe's "Marguerite" (in the jewel scene in Faust). A young lawyer coming by, stopped, and looking over our shoulder, said, "What is that?" "A picture of Marguerite," said we. "Oh, yes; I know," said he, "she kept a bakery in New Orleans." [He was thinking of Margaret Haugherty, the philanthropic old bakeress whose monument the citizens have erected in one of the public squares.] Fact.

---

A Doctor with His Hands Full.—Dr. H. W. Brown, of Waco, an old army associate of ours, and companion in Confederate days, during the month of January performed three operations for lithotomy: one of vesico-vaginal fistula, one for double harelip, three for hemorrhoids, one for fistula-in-ano, and in addition "caught six babies." This, besides a large general practice.

Pretty good for a cross roads stand.—(Our Waco friends will be down here with war-paint for that last remark we fear.)

---

Very Important to Manufacturers.—Those desiring to make exhibits of their wares at the coming meeting of the Texas State Medical Association, at San Antonio, (Tuesday, April 23, 24, 25 and 26th prox.) are notified that the Secretary, Dr. Daniel, has nothing to do with this feature, and applications for space in the exhibit hall should be addressed to the Secretary of the Committee of Arrangements, Dr. D. Berry, San Antonio, and not to the Secretary of the Association. The Association desires to encourage these exhibits, and ample preparation will be made for the convenience of exhibitors.

---

A TEN THOUSAND EDITION.—In June or July we will issue 10,000 copies of this JOURNAL, with a fine article on "Austin as a Health Resort," in addition to our usual large assortment of original articles, medical news and miscellany, editorials on leading topics, book reviews, etc. This edition will be mailed (in addition to our regular list) to the members of the several State Medical Associations in the North and West, and to the leading newspapers and the medical press. Applications for advertising space should be made early, as only a limited number of pages will be devoted to that purpose; terms reasonable. It is believed that in this way more doctors can be effectually reached than in any other. Circulars and pamphlets are thrown in the basket by most doctors. The edition will be very handsome, and will contain portrait of some distinguished Texas physician.

---

AND THE CRY IS: "STILL THEY COME"!—We welcome to our exchange list the Memphis Journal of the Medical Sciences. Volume 1, No. 1, has just been received. The Editorial Staff is composed of a brilliant array of talent, representing every department of Medical and Surgical Science, (with no editor in chief). The work is divided into six departments. That of Medicine and Therapeutics being edited by Drs. B. G. Henning and S. A. Rogers; that of General Surgery by Dr. W. B. Rogers; Obstetrics and Diseases of Children by Dr. Alex. Erskine; Gynecology by Dr. T. J. Crofford; Diseases of Eye and Ear by Dr. J. S. Minor. In the graceful salutatory it is announced that—

"While we partake duly of the modesty of feeling expressed by the Latin historian when he says, "Neither do I know, nor if I did know, would it become me to say whether, if in truly recording the transactions of the day, I have done a thing worth the while,'' still, we confess to an inward boast of having placed our standard high, and will leave nothing undone to reach it. Our secret, then, is yours, and by an open, frank and independent course in all matters coming before the profession, we hope to merit its support.''

Success to the fledgeling.

---

THE CENTRAL TEXAS MEDICAL ASSOCIATION will hold its next meeting at Waco, April 9th prox., at 10 o'clock a. m.

PROGRAMME.

''Malarial Hæmaturia,'' by W. A. Howard.

''Antiseptics in Obstetrics,'' by Drs. J. H. Sears and A. M. Curtis.

''Pernicious Vomiting of Pregnancy,'' by Dr. S. E. Shelton.

''Tobacco Chewing as a Preventive for Consumption,'' by Dr. J. W. Cock.

By special request of the president, Dr. Robt. Battey, of Rome, Georgia, will furnish a paper on ''Antiseptics in Obstetrics,'' and Dr. J. S. Letcher, of Dallas, will read a paper on the same subject.

In answer to request for volunteer papers:

''Rupture of the Uterus,'' by Prof. W. H. Wathen, of Louisville, Ky.

''Case of Abdominal Tumor in a Child,'' by Dr. W. L. Barker, of Waco.

''Case of Occlusion of Femoral Artery,'' by Dr. R. W. Park, of Waco; and other papers have been furnished.

H. C. Ghent, M. D., is president and W. O. Wilkes, M. D., is secretary.

---

AN INSULT TO THE PROFESSION.—James Gordon Bennett, the lord of the New York Herald, tried to buy the ''Lomb Prize Essay'' entitled ''Practical Sanitary and Economic Cooking for Persons of Moderate Means''—which was awarded a prize of $500, but on being told it was not for sale—solicited the privilege of publishing it in the Herald. It was granted; and what was the disgust of Secretary Irving of the American Public Health Association, Miss Abel, Mr. Lomb and all hands, to see it come out headed "The Devil sends a cook, but the Herald sends forth an antidote," "Indigestion knocked out," "Girls, the road to a man's heart is through his stomach," "An Abel Essay, and "Miss Mary Herriman Abel took the cake" etc !!!

On seeing this startling heading Secretary Watson wrote the following to Mr. Bennett, but has never received any reply to it; the presumption is that Bennett has gone off and kicked himself to death, or has gotten some other jackass to do it for him:

CONCORD, N. H., January 31, 1889.

NEW YORK HERALD, New York City :

GENTLEMEN :—The manner in which the Lomb Prize Essay appeared in the Sunday Herald of the 27th inst. is an insult to this Association, to the Committee of Award, to those who competed for the prize, and to the public. It was distinctly understood that the essay should appear in full, but, instead, you have mutilated it in an unwarrantable manner; interpolated not only a cheap and disgusting heading and uncalled for and undignified sub-headings, but even slang expressions.

Your course reflects such a degree of mediocrity upon the American Public Health Association that a statement of the facts will be publicly made at once by the Association, unless the Herald fully removes the gross misrepresentation it has made.

The fifty copies of the Herald, sent in response to an order for that number containing the Lomb Prize Essay, await your order, since, not containing the same, the Association has no use for them.

Very truly yours,

[Signed] IRVING A. WATSON, Secretary.

---

ARBOR DAY.—Texas has appointed the 22nd February, Washington's birthday, as Arbor Day. In this connection we wish to say to all who propose to plant trees or set hedges, or beautify their property in any way by tree or shrub, that Geo. Pinney, of Evergreen, Wisconsin, is the largest dealer in that line, and will sell you any kind of tree, big or little, by the tens, hundreds or thousands, at ridiculously low prices. Send for his catalogue and inform him that we asked you to do so. We have one, and it is full of new things.

